# Dynamic Contrast-Enhanced Magnetic Resonance Imaging of Regional Nodal Metastasis in Nasopharyngeal Carcinoma: Correlation with Nodal Staging

**DOI:** 10.1155/2017/4519653

**Published:** 2017-05-29

**Authors:** Bingsheng Huang, Dora Lai-Wan Kwong, Vincent Lai, Queenie Chan, Brandon Whitcher, Pek-Lan Khong

**Affiliations:** ^1^National-Regional Key Technology Engineering Laboratory for Medical Ultrasound, Guangdong Key Laboratory for Biomedical Measurements and Ultrasound Imaging, School of Biomedical Engineering, Health Science Centre, Shenzhen University, Shenzhen, China; ^2^Department of Clinical Oncology, The University of Hong Kong, Pokfulam, Hong Kong; ^3^Department of Diagnostic Radiology, The University of Hong Kong, Pokfulam, Hong Kong; ^4^Philips Healthcare, Sheung Wan, Hong Kong; ^5^Mango Solutions, London, UK

## Abstract

**Objective:**

To determine if the perfusion parameters by dynamic contrast-enhanced magnetic resonance imaging (DCE-MRI) of regional nodal metastasis are helpful in characterizing nodal status and to understand the relationship with those of primary tumor of nasopharyngeal carcinoma (NPC).

**Materials and Methods:**

Newly diagnosed patients imaged between August 2010 and January 2014 and who were found to have enlarged retropharyngeal/cervical lymph nodes suggestive of nodal disease were recruited. DCE-MRI was performed. Three quantitative parameters, *K*^trans^, *v*_e_, and *k*_ep_, were calculated for the largest node in each patient. Kruskal-Wallis test was used to evaluate the difference in the parameters of the selected nodes of different N stages. Spearman's correlation was used to evaluate the relationship between the DCE-MRI parameters in nodes and in primary tumors.

**Results:**

Twenty-six patients (7 females; 25~67 years old) were enrolled. *K*^trans^ was significantly different among the patients of N stages (N1, *n* = 3; N2, *n* = 17; N3, *n* = 6), *P* = 0.015. Median values (range) for N1, N2, and N3 were 0.24 min^−1^ (0.17~0.26 min^−1^), 0.29 min^−1^ (0.17~0.46 min^−1^), and 0.46 min^−1^ (0.29~0.70 min^−1^), respectively. There was no significant correlation between the parameters in nodes and primary tumors.

**Conclusion:**

DCE-MRI may play a distinct role in characterizing the metastatic cervical lymph nodes of NPC.

## 1. Introduction

Nasopharyngeal carcinoma (NPC) is an aggressive head and neck cancer with a high incidence in Southern China including Hong Kong. Accurate staging using the International Union Against Cancer (UICC) tumor-node-metastasis (TNM) staging system is critical for treatment planning and the prediction of patient outcome [1–3]. Regardless of the status of the primary lesion, nodal metastasis is a significant prognostic factor for survival [4]. Hence the accurate detection and the characterization of metastatic nodes are of paramount importance in NPC patient management.

DCE-MRI is a functional imaging modality that has the potential to characterize perfusion and microcirculation and, thus, may have a role to play as a noninvasive biomarker of cancer. The three quantitative parameters *K*^trans^, *v*_e_, and *k*_ep_ derived by DCE-MRI are frequently used. *K*^trans^ (in minute^−1^) is the volume transfer constant of contrast agent from blood plasma to extravascular extracellular space (EES) reflecting both blood plasma flow and permeability, *v*_e_ is the volume of EES per unit volume of tissue, and *k*_ep_ (in minute^−1^) is the flux rate constant of contrast agent from EES to plasma and equal to *K*^trans^/*v*_e_ [5]. Studies have found DCE-MRI to be useful in differentiating diseased nodes from normal nodes in head and neck squamous cell cancer, breast cancer, and cervical cancer [6–8]. In these studies, it has been shown that, in malignant nodes, microvascular permeability and the extravascular extracellular space are increased.

In our previous study we have reported the feasibility of applying DCE-MRI in NPC [9]. Our findings suggest that the evaluation of DCE-MRI by both semiquantitative and quantitative methods is useful in characterizing the neovasculature and permeability of NPC tumors. However only primary NPC tumors were studied. In the present study, we have included the evaluation of regional metastatic nodes using DCE-MRI. Since previous studies have reported increased microvascular permeability in tumors or diseased nodes (Padhani et al., 2000; Yao et al., 2011; Chang et al., 2008), we hypothesized that nodal DCE-MRI parameters, which reflect the microvascular permeability in regional metastatic lymph nodes, correlate with nodal stage and the DCE-MRI parameters in the primary tumor which is an indicator of tumor aggressiveness.

## 2. Materials and Methods

### 2.1. Patients

This study was approved by the Institutional Review Board of The University of Hong Kong/Hospital Authority Hong Kong West Cluster. All consecutive newly diagnosed NPC patients referred to the MRI Unit at The University of Hong Kong between August 2010 and January 2014 were prospectively included. Patients with presumed metastatic regional lymph nodes, based on enlarged lymph nodes (>1 cm short axis diameter) on MRI, and supported by increased metabolic activity on ^18^F-FDG PET-CT scans (nodal SUVmax > 2.5) performed within one week of the MRI scans, were included. The final diagnosis of metastatic lymph nodes and the N stage were achieved by both the imaging findings (MRI and PET-CT) and histological results. Written informed consent was obtained for all patients. TNM staging was performed according to the Joint Committee on Cancer (AJCC) staging system [10].

### 2.2. DCE-MRI Techniques

After routine structural MRI acquisition, DCE-MRI of the nasopharynx and upper neck was performed on a 3.0-T MRI system (Achieva; Philips Healthcare). Four acquisitions were obtained in a chronological order with a field of view (FOV) of 22 × 22 × 6 cm (AP × RL × FH): precontrast T_1_-weighted fast field echo (FFE) acquisition using a flip angle of 5° (“FA5” acquisition) in 1 minute 22 seconds; T_2_-weighted imaging (“T2W” acquisition) in 50 seconds; B1 mapping measurement acquisition (“B1MAP” acquisition) in 1 minute 23 seconds; and DCE acquisition using a flip angle of 15° (“FA15” acquisition) in 6 minutes 47 seconds with 65 dynamic scans. The details of scanning protocols have been described in our previous paper [9]. All four acquisitions were performed in the same anatomical region and reconstructed to the same resolution. The contrast agent Gd-DOTA (Dotarem, Guerbet, France) was injected intravenously as a bolus into the blood at around the 8th dynamic acquisition using a power injector system (Spectris Solaris, MedRad, USA), immediately followed by a 25-mL saline flush at a rate of 3.5 mL per second. The dose of Gd-DOTA was 0.1 mmol/(kg body weight) for each patient.

### 2.3. Data Analysis

All the acquired DCE-MRI images were used for quantitative analysis, and the parametric maps of *K*^trans^, *k*_ep_, and *v*_e_ were calculated as in our previous publication [9]. The procedure of calculating parametric maps of *K*^trans^, *k*_ep_, and *v*_e_ was performed using the software dcemriS4 http://cran.r-project.org/web/packages/dcemriS4/) developed by Whitcher and Schmid [11]: firstly the maps of contrast concentration of 65 time points were calculated from the DCE-MRI images and secondly for each voxel the contrast concentration curve and the population AIF [9] were fitted to the pharmacokinetic model to calculate the maps of the three parameters.

For each patient, since there are usually more than 1 metastatic node, as done in the literature [12–14], the largest metastatic node within the scanned region determined by the sum of long and short of axis was identified by a neuroradiologist (PL Khong) based on conventional anatomical MR images (T1-weighted, T2-weighted, and postcontrast T1-weighted) and treated as the representative node in our study. The node boundary was identified in the relevant consecutive slices of the T2W images of DCE-MRI scan and a series of two-dimensional regions of interest (ROI) were contoured using the software ImageJ (NIH, USA) (V Lai). The average *K*^trans^, *k*_ep_, and *v*_e_ values in each node were calculated and used for further analysis.

The normality of the DCE-MRI parameters distribution in our cohort was checked using Shapiro–Wilk test. ANOVA (for data of normal distribution) or Kruskal-Wallis test (for data which are not normal distribution) were used to evaluate the difference among tumor N stages. Pearson's correlation (for data of normal distribution) or Spearman's correlation (for data which are not normal distribution) was performed to study the correlations between the DCE-MRI parameters in nodes and in primary tumors and between the DCE-MRI parameters in nodes and nodal size. All statistical analyses were performed using SPSS 20 (SPSS Inc, Chicago, IL, USA), and *P* < 0.05 was considered statistically significant.

## 3. Results

The cohort characteristics of this study are shown in Table 1. A total of 26 patients were included and 7 of them were female. The mean age was 45 years (range, 25~67 years; SD, 12 years). The correlations between the DCE-MRI parameters in nodes and nodal size are insignificant with all *P* values higher than 0.2.

**Figure 1:**
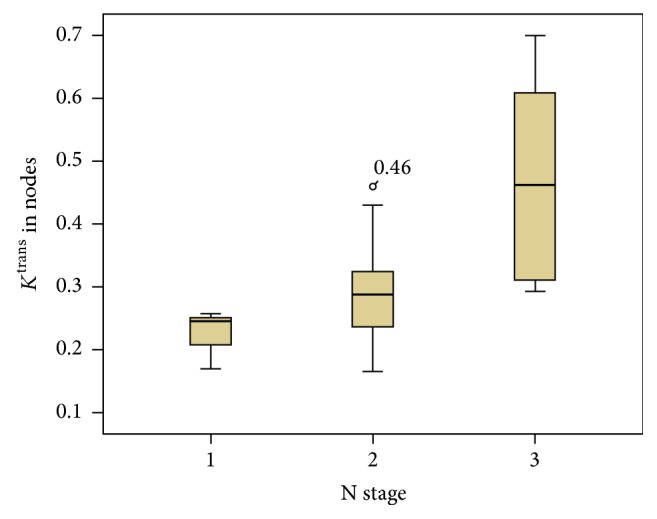
Box plots showing significant difference of *K*^trans^ in metastatic nodes by Kruskal-Wallis test among N staging (*P* = 0.015).

By Kruskal-Wallis test, *K*^trans^ in the largest node of each patient was significantly different among the various N stages (*P* = 0.015). Median values and ranges were N1 (*n* = 3), 0.24 min^−1^ and 0.17~0.26 min^−1^; N2 (*n* = 17), 0.29 min^−1^ and 0.17~0.46 min^−1^; N3 (*n* = 6), 0.46 min^−1^ and 0.29~0.70 min^−1^, respectively (Figure 1). *k*_ep_ and *v*_e_ in the largest node were not correlated with N stage (*P* = 0.485 and 0.113, resp.). The median values and ranges of *k*_ep_ were N1, 0.44 min^−1^ and 0.33~0.66 min^−1^; N2, 0.38 min^−1^ and 0.13~0.67 min^−1^; N3, 0.36 min^−1^ and 0.10~0.63 min^−1^, respectively. The median values and ranges of *v*_e_ were N1, 0.32 and 0.29~0.70; N2, 0.39 and 0.32~0.60; N3, 0.40 and 0.24~0.62, respectively (Table 2).

**Figure 2:**
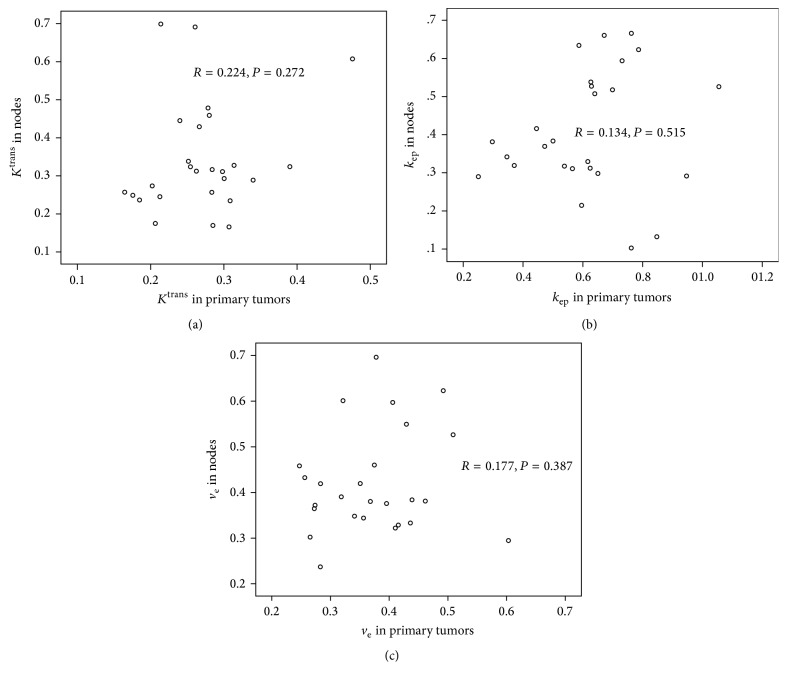
Scatter plots show no significant correlations between the DCE-MRI parameters in metastatic nodes and in primary tumors by Spearman's correlation: (a) *K*^tran^; (b) *k*_ep_; (c) *v*_e_.

The mean values and ranges of *K*^trans^, *k*_ep_, and *v*_e_ in the primary tumors were 0.27 min^−1^ and 0.16~0.48 min^−1^; 0.62 min^−1^ and 0.25~1.06 min^−1^; 0.37 and 0.25~0.60, respectively. Using Spearman's correlation, none of the three DCE-MRI parameters in nodes were significantly correlated with the corresponding parameters in the primary tumors: for *K*^trans^, *r* = 0.224 and *P* = 0.272; for *k*_ep_, *r* = 0.134 and *P* = 0.515; for *v*_e_, *r* = 0.177 and *P* = 0.387 (Figure 2).

## 4. Discussion

In this study, we evaluated the correlation between the DCE-MRI parameters in the metastatic lymph nodes and tumor N stage. Studies have been performed to compare the DCE-MRI parameters in lymph nodes that were confirmed to be positive and negative for malignancy by histology [15, 16]. Such studies have showed that malignant nodes have higher vascularity and microvessel permeability compared to benign nodes. Similarly, our findings also support the hypothesis that increased vascular permeability reflected by *K*^trans^ in metastatic lymph nodes is positively correlated with N stage; that is, the higher the N stage, which is an indicator of aggressiveness and portends poorer prognosis [4, 17], the higher the *K*^trans^, which reflects higher permeability and perfusion. Such significant correlation should not be due to the nodal size, since the correlation between these perfusion parameters and nodal size was insignificant. It is widely accepted that these perfusion and permeability characteristics reflected by DCE-MRI are directly related to the tumor angiogenic activity [18–20] and that tumor N staging reflects the spread and extent of lymph node metastasis [10]. Based on the fact that angiogenesis is required to support cancer growth and metastasis, our results may be explained by the fact that nodes with more angiogenesis facilitate the spread of cancer cells in lymph node chains leading to a higher N stage.

We did not observe any significant difference in *v*_e_ or *k*_ep_ among tumors of different N stages. The *k*_ep_, calculated as *K*^trans^/*v*_e_, is not an independent parameter; *v*_e_ is the measurement of volume of EES per unit volume of tissue and reflects the available space for contrast permeability. The lack of significance in the difference between *v*_e_ may indicate that the higher *K*^trans^ may be due mainly to the increased blood flow (perfusion) but not the EES volume, and that the increase in *K*^trans^ may precede the increase in EES volume. Further prospective studies are required to confirm this finding.

The present study found that the DCE-MRI parameters in nodes were not significantly correlated with the corresponding parameters in primary tumors, similar to another study performed in cervical cancers and its nodal metastases [8]. This suggests that the tumor microvessel environment, that is, perfusion and vascular permeability, in the primary NPC tumor is independent of the characteristics in its metastatic lymph nodes. Thus, tumors with high perfusion and vascular permeability may neither develop nodal metastasis nor have more metastatic nodes. This discrepant finding between the primary tumor and its metastasis suggests heterogeneity that is intermetastatic and supports the notion that the metastasizing process of a malignant primary tumor may be related to genetic alterations in the primary tumor, which may be heterogeneous in nature [21, 22]. The primary tumor may release a number of cells into the circulation; however only a small fraction of these cells establish metastases in a favourable organ or node in a nondeterministic manner [23]. Thereafter, continual evolution of the primary tumor reflects local selective advantages rather than future selective advantages, and thus growth at metastatic sties is not dependent on additional genetic alterations in the primary tumor. Such discrepancy between metastatic nodes and primary tumor indicates that attention should also be needed to the study of the metastases.

The results of our study, although the mechanism of which is still unclear, may have some clinical implications for clinical management of NPC patients. Since the perfusion parameters in nodes, but not in primary lesion, were significantly correlated with the tumor N stage, clinicians may pay more attention to these parameters in metastatic nodes due to the fact that N staging is critical in prognosis. On the other hand, one may expect to evaluate the role of such perfusion parameters, which reflect the functional activity in tumors and metastatic nodes, in NPC patient management in the future studies.

Our study has some limitations. Firstly, the MRI scan coverage included the primary tumor and upper neck but not the entire neck. Thus, some regional metastatic lymph nodes may not have been included. However, it has been reported that lymph node metastasis generally spreads from the upper neck to the lower neck [24–26]; therefore the first lymph node station of spread is in the locoregional node adjacent to the primary tumor. Secondly, there may be an element of error in the placement of the ROI, as this procedure was completed manually, although accuracy was improved by confirming the location of the ROIs on the coregistered conventional T2W images.

## 5. Conclusion

In a cohort of new NPC patients, we found that perfusion and permeability based on DCE-MRI are higher in regional nodes of higher N stage tumors, and that the parameters in the nodes have no relationship with the corresponding parameters in its primary tumor. These findings showed that DCE-MRI in the metastatic lymph plays a distinct role in characterizing the nodal status in NPC. This finding, if further verified, may have important impact in the staging and management of NPC patients with metastatic lymph nodes.

## Figures and Tables

**Table 1:** Patient demographic data and tumor characteristics (*N* = 26).

*Baseline characteristics*	
Age (years)	
Range	25~67
Median	45
Mean ± SD	45 ± 12
Sex	
Number of female patients	7
Number of male patients	19
*Stage*	
T stage	Number of patients
1	10
2	5
3	9
4	2
N stage	Number of patients
1	3
2	17
3	6
M stage	Number of patients
0	25
1	1

*Notes*. Age (years)is patient age at diagnosis; T, N, and M stages were evaluated according to the American Joint Committee on Cancer (AJCC) staging system.

Median values and ranges were N1 (*n* = 3), 0.24 min^−1^ and 0.17~0.26 min^−1^; N2 (*n* = 17), 0.29 min^−1^ and 0.17~0.46 min^−1^; N3 (*n* = 6), 0.46 min^−1^ and 0.29~0.70 min^−1^, respectively (Figure 1). *k*_ep_ and *v*_*e*_ in the largest node were not correlated with N stage (*P* = 0.485 and 0.113, resp.). The median values and ranges of *k*_ep_ were N1, 0.44 min^−1^ and 0.33~0.66 min^−1^; N2, 0.38 min^−1^ and 0.13~0.67 min^−1^; N3, 0.36 min^−1^ and 0.10~0.63 min^−1^, respectively. The median values and ranges of* v*_*e*_ were N1, 0.32 and 0.29~0.70; N2, 0.39 and 0.32~0.60; N3, 0.40 and 0.24~0.62, respectively.

**Table 2:** The perfusion parameters of metastatic nodes among N stages (*n* = 26).

N stage	*K* ^trans^ (min^−1^)	*k*_ep_ (min^−1^)	*v*_e_
N1 (*n* = 3)	0.24 (0.17~0.26)	0.44 (0.33~0.66)	0.32 (0.29~0.70)
N2 (*n* = 17)	0.29 (0.17~0.46)	0.38 (0.13~0. 67)	0.39 (0.32~0.60)
N3 (*n* = 6)	0.46 (0.29~0.70)	0.36 (0.10~0.63)	0.40 (0.24~0.62)

## Abbreviations

NPC: Nasopharyngeal carcinoma
TNM: Tumor-node-metastasis
EES: Extravascular extracellular space
AJCC: The American Joint Committee on Cancer
FOV: Field of view
FFE: Fast field echo
T2W: T_2_-weighted
B1MAP: B1 mapping
FA15: Flip angle of 15°
ROI: Regions of interest.
